# Epitope Fingerprinting for Recognition of the Polyclonal Serum Autoantibodies of Alzheimer's Disease

**DOI:** 10.1155/2015/267989

**Published:** 2015-08-31

**Authors:** Luiz Carlos de Oliveira-Júnior, Fabiana de Almeida Araújo Santos, Luiz Ricardo Goulart, Carlos Ueira-Vieira

**Affiliations:** ^1^Laboratório de Genética, Instituto de Genética e Bioquímica, Universidade Federal de Uberlândia, Rua Acre s/n, Bloco 2E sala 230, Campus Umuarama, 38400-902 Uberlândia, MG, Brazil; ^2^Faculdade de Medicina, Universidade Federal de Uberlândia, Avenida Para 1720, Bloco 2U sala 23, Campus Umuarama, 38400-902 Uberlândia, MG, Brazil; ^3^Laboratório de Nanobiotecnologia, Instituto de Genética e Bioquímica, Universidade Federal de Uberlândia, Rua Acre s/n, Bloco 2E sala 230, Campus Umuarama, 38400-902 Uberlândia, MG, Brazil

## Abstract

Autoantibodies (aAb) associated with Alzheimer's disease (AD) have not been sufficiently characterized and their exact involvement is undefined. The use of information technology and computerized analysis with phage display technology was used, in the present research, to map the epitope of putative self-antigens in AD patients. A 12-mer random peptide library, displayed on M13 phages, was screened using IgG from AD patients with two repetitions. Seventy-one peptides were isolated; however, only 10 were positive using the Elisa assay technique (Elisa Index > 1). The results showed that the epitope regions of the immunoreactive peptides, identified by phage display analysis, were on the exposed surfaces of the proteins. The putative antigens MAST1, Enah, MAO-A, X11/MINT1, HGF, SNX14, ARHGAP 11A, APC, and CENTG3, which have been associated with AD or have functions in neural tissue, may indicate possible therapeutic targets.

## 1. Introduction

Alzheimer's disease (AD) is the most important cause of dementia. Its prevalence increases with age and, together with increasing life expectancy, has created the expectation of an increase in the number of cases, especially in developed countries [1–4]. Due to its devastating effect on cognition and high social and economic cost [5, 6], AD has become an important subject of research and, due to its characteristics, is also a challenge. This is especially true since the neurodegenerative process may progress for many years before clear behavioral and cognitive symptoms permit diagnosis [7, 8].

Following the original description of AD in 1906, the presence of *β*-amyloid (A*β*) deposits, senile plaques (SP), and neurofibrillary tangles (NFT) has been established as key markers of the disease [9, 10]. The search for improved understanding of its development has focused on these components, which have also been linked to numerous other neurobiological processes as well as genetic and environmental factors [11]. Despite extensive research, our understanding of AD is still limited because accurate diagnosis of the onset of the disease is often not possible [12].

Soon after it was observed that SP and NFT are accompanied by an inflammatory process in the immune system, this system began to be investigated regarding its role in AD pathogenesis [13, 14]. As in other central nervous system diseases, Parkinson's disease, Lewy corpuscles dementia, and obsessive compulsive disorder, there appears to be a relationship between inflammatory processes and humoral response to AD [15, 16].

Several studies have discovered an abundant presence of antibodies directed at targets in brain neural tissue, cerebrospinal fluid, and the serum of patients with AD. Antibodies against neurotransmitter receptors (glutamate, dopamine, serotonin, and acetylcholine), enzymes (ATP synthase and aldolase), cytoskeletal proteins, and microglia have been described [17–20]. Their role in the development of Alzheimer's disease is still uncertain and may simply be the result of neuronal death from exposure to autoantigens or may have some contribution to the pathological process [21–23]. The characterization of these autoantibodies (aAb), their antigens, and their role in disease may be a means for the development of improved diagnostic tools and the identification of new therapeutic targets.

Phage display (Ph.D.) technology is useful for the identification of peptides or antibodies on the surface of the filamentous M13 bacteriophage capsid. This capsule permits exposure to an extensive diversity of peptides that can bind to various targets and be identified using peptide library techniques. This methodology has been proven useful not only for the selection of peptides that mimic proteins but also for the identification and description of epitopes recognized by antibodies [24, 25]. It also allows for the production of the monoclonal antibodies used to treat several diseases, vaccines, and diagnostic tests as well as several uses in nanotechnology [26].

Phage display findings can be analyzed with different bioinformatic tools: the identification of consensus motifs among selected sequences, the identification of possible targets by linear and conformational (3D structure) comparison with protein databanks, and assessments of their putative epitopes with their degree of antigenicity. This information can be extremely useful for planning experiments, designing drugs, and other applications [27–29].

The present study identified mimetic peptides of target antigens in the circulating IgG present in the serum of patients with AD. Our use of the phage display technique, together with bioinformatic tools, may represent one of the first evidences of the presence of autoantibodies and their putative epitope mapping, in AD.

## 2. Materials and Methods

### 2.1. Recruitment of Patients, Diagnostic Criteria, and Sample Collection

Serum samples from AD patients and healthy controls, matched by sex and age, were obtained from the University Hospital of Uberlandia. For the diagnosis of dementia, the DSM-IV TR criteria were used [30] and for the diagnosis of AD we used the criteria of the National Institute of Neurological and Communicative Disorders and Stroke/Alzheimer's Disease and Related Disorders Association (NINCDS-ADRDA) [31]. Patients were stratified according to the Clinical Dementia Rating (CDR), Portuguese version [32]. The controls were evaluated using the Mini-Mental State Exam [33]. This research was approved by the Research Ethics Committee of the Federal University of Uberlandia (number 304/09).

### 2.2. Selection of Peptides That Mimic AD Self-Antigens (Mimotopes)

The phage selection was performed using a pool of sera from AD patients and healthy (control) individuals. Immunoglobulin G (IgG) was secured using magnetic beads coupled to protein G Dynabeads (Invitrogen). For subtraction of nonspecific peptides, 10 *μ*L of the M13 phage library (PhD12, New England Biolabs Inc.; 1 × 10^11^ viral particles) was added to 190 *μ*L of TBS-Tween 0.1%. After 30 minutes of incubation, magnetic separation was performed. The phage eluate was subtracted two more times prior to the positive selection, which was performed for 30 minutes against IgG-coupled beads of AD patients, completing one selection cycle. This procedure was repeated twice. Finally, bound phages were recovered from the beads by acid elution (500 *μ*L of glycine, pH 2) for 10 minutes and then neutralized with 75 *μ*L of Tris (pH 9).

Selected phages were amplified, purified, and titrated according to the Ph.D. Phage Display Libraries Instruction Manual (New England Biolabs).

### 2.3. DNA Extraction and Sequencing

After three rounds of selection, 96 blue colonies were randomly selected and their phage single strand DNA was isolated using iodide buffer extraction procedures [34].

### 2.4. Bead-ELISA (Enzyme Linked Immunosorbent Assay in Bead)

The selected peptide-phage clones were used in the bead-ELISA assay against IgG from controls and AD patients to evaluate their reactivity and specificity.

Fifty microliters of phage supernatant was incubated with IgG coupled in magnetic beads (Invitrogen) for one hour with stirring, at room temperature. Using a magnetic apparatus, the microspheres were precipitated, washed six times with TBS-T 0.1%, and incubated with monoclonal anti-M13 peroxidase conjugate (GE Healthcare) diluted 1 : 5000 in TBS-T 0.1% and 5% BSA for one hour with stirring, at room temperature. Microspheres were again precipitated and washed six times and the reaction was observed with buffer orthophenylenediamine (OPD) to 1 mg/mL plus 3% hydrogen peroxide (H_2_O_2_). The results were expressed as an arbitrary ELISA Index (EI) and calculated as follows: EI = Abs of serum sample/cut-off, where the cut-off was determined as the mean absorbance of the negative control sera plus two standard deviations. Values of EI > 1.0 were considered positive.

### 2.5. Bioinformatics

**Figure 1:**
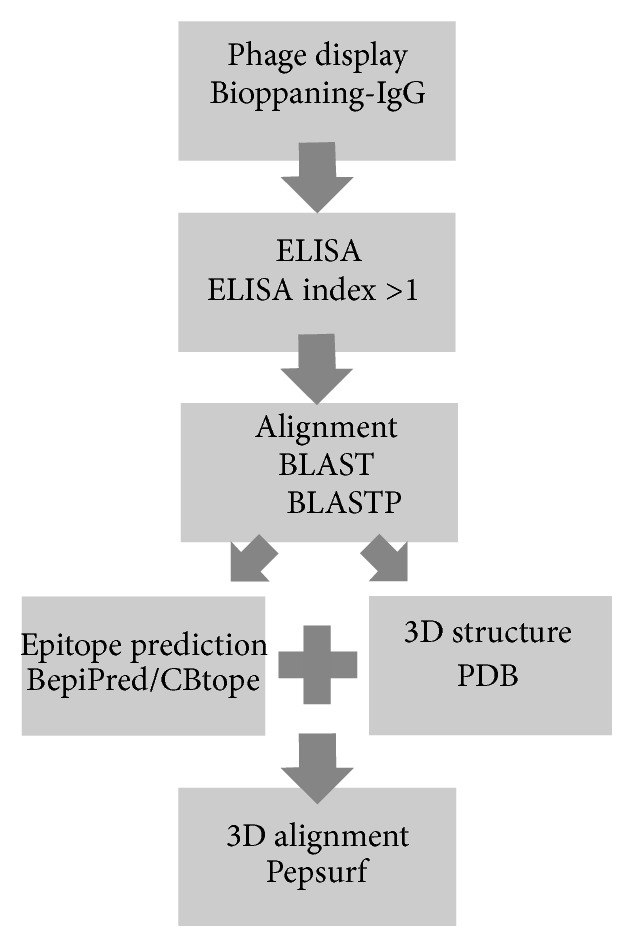
Bioinformatics workflow.

The vector sequences were removed and the deductions of peptide sequences were performed using the ExPASy Translate Tool (http://web.expasy.org/translate). Afterwards, the peptide sequences were submitted to* in silico *analysis (Figure 1).

For a more detailed analysis, the sequence of positive peptides selected by the ELISA assay (EI > 1) was subjected to alignment using the BLAST tool (http://blast.ncbi.nlm.nih.gov/) and compared with those available in the database of nonredundant protein sequences using the BLASTP algorithm, limiting the search to* Homo sapiens* sequences.

The proteins indicated in alignment were selected for the next step of the analysis. We excluded unnamed sequences which had only been predicted or that were from unknown proteins. Those sequences with low *E*-value were analyzed to determine whether the region of alignment with the peptide was a predicted epitope using BepiPred (http://www.cbs.dtu.dk/services/BepiPred/) [35] for linear B-cell epitopes and CBTOPE (http://www.imtech.res.in/raghava/cbtope/) for conformational B-cell epitopes [36]. If the region of alignment was found to be a predicted epitope and the three-dimensional structure was available in a protein databank format (http://www.rcsb.org/pdb/home/home.do), this protein was selected for the next step of analysis.

The program PEPSURF (http://pepitope.tau.ac.il/) was used to map the putative mimotope selected by phage display in the three-dimensional protein structure of the protein [29].

### 2.6. Statistical Analysis

Statistical analysis was performed using the GraphPad Prism version 5.00 (GraphPad Software Inc.).

## 3. Results

In this study 100 patients who had registered cognitive disorders were evaluated. Only 10 of these patients had complete AD diagnosis with laboratory tests, imaging, and assessment of cognitive function by neuropsychological tests. As paired healthy control (HC), we used 10 cognitively healthy individuals.

**Figure 2:**
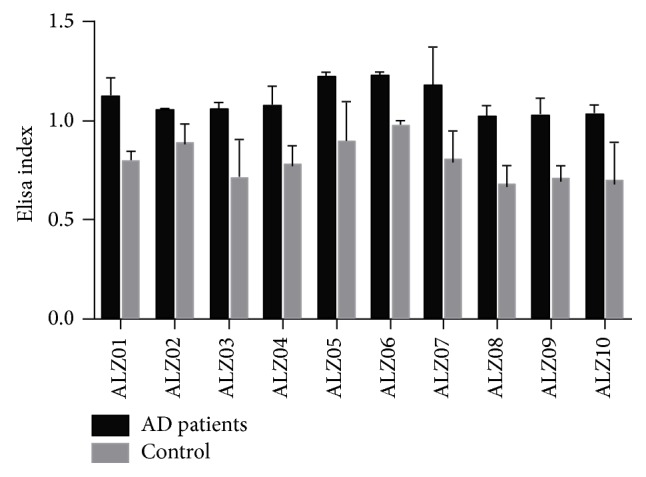
Detection of IgG antimimotope in serum from patients with Alzheimer's disease by Elisa using peptide-phage selected by phage display. Values of EI > 1.0 were considered positive (Student's* t*-test, *P* < 0.05). For clarity, peptides with Elisa Index values lower than 1 were omitted.

Phage display selection of a 12-mer random peptide library generated 75 peptides, of which 71 were distinct sequences. A phage ELISA assay was performed with these clones using a pool of serum from the patients and from the controls. The result showed that of the 71 peptides, only 10 were highly reactive mimotopes when compared with the controls (IE > 1). This suggested that circulating IgG from AD patients recognizes these specific peptides (Figure 2).

Those peptides with distinct sequences were subsequently chosen for in-depth characterization through bioinformatics. The data are presented in Table 1. As can be seen, only nine sequences of peptides led to the identification of targets according to established criteria for the bioinformatics analysis.

**Figure 3:**
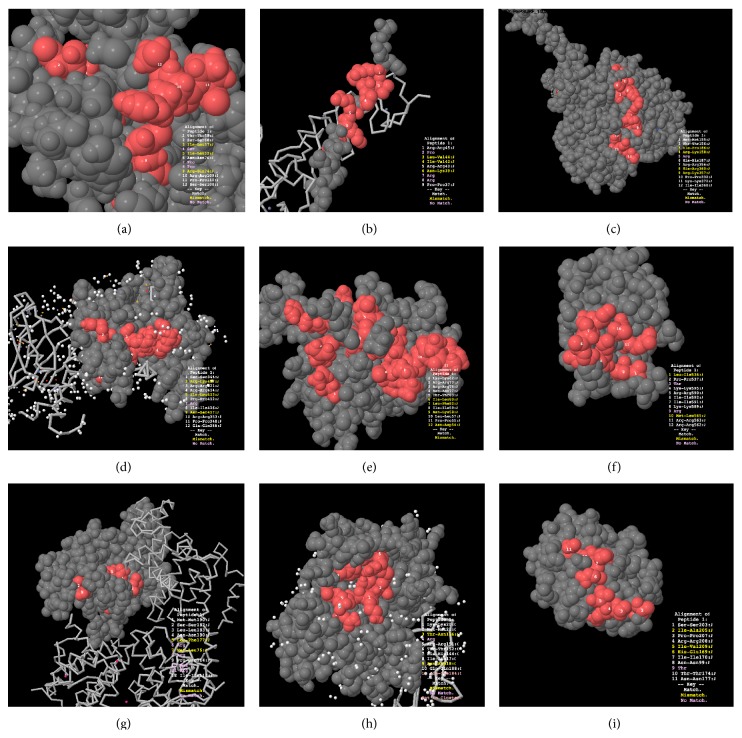
Three dimensional epitope prediction using the PepSurf program. The peptide alignment regions are shown in red. All of the peptides align with external regions. (a) MAST1; (b) Enah; (c) MAO-A; (d) X11/MINT1; (e) HGF; (f) SNX14; (g) ARHGAP 11A; (h) APC; (i) CENTG3. Source: Martz E. FirstGlance in Jmol (http://firstglance.jmol.org).

After the initial identification of targets for alignment and prediction of linear and structural epitopes, the three-dimensional alignment, using the PepSurf program, was performed. This result demonstrated that peptide sequences from phage display were mapped in exposed regions (external surfaces) of target proteins and could be accessible to antibodies (Figure 3).

## 4. Discussion

Phage display technology can be considered a subtractive proteomic strategy for the selection of specific molecules without known targets. This is due to its combinatorial nature, favoring the random binding to several molecules. It is, for this reason, an important tool for the identification of biomolecules because it exposes a large variety of ligands to many targets at the same time and requires only minimal knowledge of the starting proteome/immunome target [37]. However, this technology has a great disadvantage: only linear or simple cyclic peptides can be incorporated into phage pIII protein [38].

Since there was the possibility of the phage binding on components of the screening system such as plastic, magnetic bead, protein G [39], or irrelevant IgG, we performed a subtractive selection twice using IgG from a binding assay of healthy controls before selection with IgG from Alzheimer's patients. This precaution was taken to avoid the selection of peptides binding in the background.

Our selection and analysis strategy resulted in the identification of ten potential mimotopes recognized by the IgG present in the serum of patients with AD. It was possible to select peptides by phage display and prevalidate them as potential new products for specific diagnosis of thyroid cancer [34], neurocysticercosis [40, 41], leishmaniasis, dengue, and leprosy [42]. The peptides selected in this work are also new potential tools for developing specific serum diagnostics for AD. Evaluation with large samples will be necessary for validation in serum platforms such as the ELISA assay.

Autoantibodies are important for AD progression. Patients with AD have a low titer of serum levels of the anti-beta-amyloid antibodies (A*β*1–42, A*β*1–15 and A*β*16–30) compared with age matched non-AD controls [43]. Antibodies against A*β* have potential use in AD treatment [44, 45]. However, the action of the autoantibodies on other neuroproteins is still unclear.

The putative epitopes of the self-antigens, using the mimotopes, were mapped and are presented in Table 2. The target proteins have an important function in the central nervous system or are involved in AD (Table 2).

The ALZ01 peptide is aligned with the MAST1 sequence. MAST1 is a member of the microtubule associated serine/threonine kinase family [35]. It is an important component of the postsynaptic region [46] and one of the differentially expressed genes in the brain of patients with AD [47].

The Enah or Mena proteins, with which peptide ALZ02 is aligned, are a component of the neural growth cone [48], important for neural development [49] and axonal structure [50].

Alz03 is a putative mimotope of MAO-A, an important enzyme of the catecholamine pathway. Some studies have shown changes of the catecholamine in AD. NE levels are decreased in the hippocampus of patients with AD [51]. In addition, specific variants of PS1, an important enzyme in the formation of Ab, could influence the catalytic activity of MAO-A [52].

The ALZ04 peptide sequence is aligned with the X11 protein family. These proteins, also known as Mints or APBA (APP binding family A), are multidomain adaptor proteins [53]. They are involved in many cellular processes important for neuronal function including the regulation of ion channel function, cellular traffic, synaptic vesicle docking, and exocytosis. The X11s proteins are also involved in APP processing [54, 55]. The Mint1 PTB domain interacts with APP, regulating its traffic. Several studies have shown that gene deletion or suppression interferes with the Ab levels [56].

Another interesting mimotope of a putative self-antigen identified in the present research was the HGF. This polypeptide is a growth factor that acts like a semaphorin in the neural development [57]. Some studies have shown that levels of HGF are increased in the cerebrospinal fluid of patients with AD [58]. Also, in the brain of patients with AD, there is an increase in HGF expression which may indicate a response to injury [59].

The SNX14 protein, also mapped by the peptides from phage display, is an important element for endocytosis and endosomal signaling [60]. It has been shown, in mice, that it regulates the intrinsic excitability of pyramidal neurons [61]. The SNX12, another family member of nexins sorting, is involved in the development of the cerebral cortex [62] and regulates the endocytosis of BACE-1 [63]. Its levels are diminished in the brains of AD patients [64].

The APC protein, in mice, was found to reduce the production of Ab. The mechanism involved appears to be a stimulation of the alpha secretase activity [65]. The intracerebral infusion of APC also reduced the excitotoxicity mediated by NMDA receptors [66].

The CENTG3 antibody, also known as AGAP3 and mapped by our mimotopes, is important for AMPA receptor traffic to the neural membrane during long term potentiation, which strengthens the synapse [67]. An alternative splicing variant of AGAP3, CRAG, acts like a semaphorin [68].

The involvement of aAb in neurodegenerative diseases can be varied and uncertain. Antibodies can act as receptor agonists or antagonists, coagonists, activate the complement proteins, or lead to internalization of receptors [15]. These aAb can act directly in the disease process or may be formed only as a consequence of the exposure to new antigens by neuronal death. But, interestingly, they may also become potential biomarkers to improve understanding of AD biology and progression.

The role of aAb in AD has not been determined despite frequent descriptions of its presence in the serum and cerebrospinal fluid of AD patients. Some aAb, such as that generated against A*β*, appear to prevent the deposition and formation of fibrils and plaques by reducing neurotoxicity. Their presence in healthy individuals suggests a homeostatic role [22, 23].

Nagele et al. [69] identified several self-antigens through protein microarrays by demonstrating the presence of various aAb in the serum of patients with AD. The antigens with higher aAb reactivity were suggested as potential biomarkers. Many of the putative self-antigens identified in this study have a clear involvement with AD but the effective participation of these aAb in AD is still to be determined.

The use of mimic peptide as a diagnostic, rather than full protein, may yield increases in the specificity of the* in vitro* reaction. Since only the reactive region of the biomarker target will be in contact with the antibody, the background reactions tend to be low. Further studies with large sample sizes are necessary to define the potential of the mimotope peptide here isolated as a new biomarker.

## 5. Conclusion

The combination of* in silico *approaches and phage display technology was found to be an important tool in the identification of putative novel targets in Alzheimer's disease. The success of our epitope fingerprinting was based on a strategy that involved performing a subtractive selection against the IgG of AD patients. We identified mimotopes that mimic self-antigens and these mimotopes were recognized by the autoantibodies of AD patients, which may indicate potential usefulness in the diagnosis of this disease.

## Figures and Tables

**Table 1:** Peptide sequence and position of alignment in putative Alzheimer's disease self-antigens.

Clone	Peptide sequence	Alignment region	Putative protein matched	PDB	Accession number NCBI
ALZ01	TSISINPPRRPS	672–683	MAST1	2M9X	AAH27985.2
ALZ02	SRPRPLIRNRRP	341–350	Enah	2XQN	AAH65238.1
ALZ03	MTIRRHRHRPKI	128–131	MAO-A	2Z5Y	P21397.1
ALZ04	SRRRIPRINRPQ	431–438	X11/MINT1	1X11	Q02410.3
ALZ05	KRRNTILINLPN	4–9	HGF	2HGF	P14210.2
ALZ06	TPIKKMIRRLPH	—	—	—	—
ALZ07	LPTKRIIKRMRR	502–508	SNX14	4BGJ	Q9Y5W7.3
ALZ08	MSLNLRMRPMRI	449–453	ARHGAP 11A	3EAP	Q6P4F7.2
ALZ09	KMTRRTHINQIS	111–115	APC	1AUT	1AUT_C
ALZ10	RSIPRIHINTTN	235–246	CENTG3	3IHW	3IHW_A

**Table 2:** Identity of the self-antigens mapped by mimotopes.

Database ID	Description	Protein
AAH27985.2	Microtubule associated serine/threonine kinase 1	MAST1
AAH65238.1	Enabled homolog (Drosophila)	ENAH
P21397.1	Monoamine oxidase A	MAOA
Q02410.3	Amyloid beta (A4) precursor protein-binding, family A, member 1	APBA1
P14210.2	Hepatocyte growth factor (hepapoietin A; scatter factor)	HGF
Q9Y5W7.3	Sorting nexin 14	SNX14
Q6P4F7.2	Rho GTPase activating protein 11A	ARHGAP11A
NM_000312	Protein C (inactivator of coagulation factors Va and VIIIa)	PROC
AF413079.1	Homo sapiens centaurin gamma 3 mRNA	CENTG3

## References

[1] Brunnström H., Gustafson L., Passant U., Englund E. (2009). Prevalence of dementia subtypes: a 30-year retrospective survey of neuropathological reports. *Archives of Gerontology and Geriatrics*.

[2] Ferri C. P., Prince M., Brayne C. (2005). Global prevalence of dementia: a Delphi consensus study. *The Lancet*.

[3] Hebert L. E., Scherr P. A., Bienias J. L., Bennett D. A., Evans D. A. (2003). Alzheimer disease in the US population: prevalence estimates using the 2000 census. *Archives of Neurology*.

[4] Rocca W. A., Hofman A., Brayne C. (1991). Frequency and distribution of Alzheimer's disease in Europe: a collaborative study of 1980–1990 prevalence findings. *Annals of Neurology*.

[5] Olesen J., Gustavsson A., Svensson M. (2012). The economic cost of brain disorders in Europe. *European Journal of Neurology*.

[6] Jönsson L., Jönhagen M. E., Kilander L. (2006). Determinants of costs of care for patients with Alzheimer's disease. *International Journal of Geriatric Psychiatry*.

[7] Fox N. C., Schott J. M. (2004). Imaging cerebral atrophy: normal ageing to Alzheimer's disease. *The Lancet*.

[8] Wilson R. S., Leurgans S. E., Boyle P. A., Bennett D. A. (2011). Cognitive decline in prodromal Alzheimer disease and mild cognitive impairment. *Archives of Neurology*.

[9] Braak E., Griffing K., Arai K., Bohl J., Bratzke H., Braak H. (1999). Neuropathology of Alzheimer's disease: what is new since A. Alzheimer?. *European Archives of Psychiatry and Clinical Neuroscience*.

[10] Jack C. R., Petersen R. C., Xu Y. (1998). Rate of medial temporal lobe atrophy in typical aging and Alzheimer's disease. *Neurology*.

[11] Selkoe D. J. (2001). Alzheimer's disease: genes, proteins, and therapy. *Physiological Reviews*.

[12] Gustaw-Rothenberg K., Lerner A., Bonda D. J. (2010). Biomarkers in Alzheimers disease: past, present and future. *Biomarkers in Medicine*.

[13] Bouras C., Riederer B. M., Kövari E., Hof P. R., Giannakopoulos P. (2005). Humoral immunity in brain aging and Alzheimer's disease. *Brain Research Reviews*.

[14] Khandelwal P. J., Herman A. M., Moussa C. E.-H. (2011). Inflammation in the early stages of neurodegenerative pathology. *Journal of Neuroimmunology*.

[15] Diamond B., Huerta P. T., Mina-Osorio P., Kowal C., Volpe B. T. (2009). Losing your nerves? Maybe it's the antibodies. *Nature Reviews Immunology*.

[16] Maetzler W., Berg D., Synofzik M. (2011). Autoantibodies against amyloid and glial-derived antigens are increased in serum and cerebrospinal fluid of lewy body-associated dementias. *Journal of Alzheimer's Disease*.

[17] Daniilidou M., Tsolaki M., Giannakouros T., Nikolakaki E. (2011). Detection of elevated antibodies against SR protein kinase 1 in the serum of Alzheimer's disease patients. *Journal of Neuroimmunology*.

[18] Davydova T. V., Voskresenskaya N. I., Gorbatov V. Y., Fomina V. G., Doronina O. A., Maksunova I. V. (2009). Production of autoantibodies to glutamate during Alzheimer's dementia. *Bulletin of Experimental Biology and Medicine*.

[19] Levin E. C., Acharya N. K., Han M. (2010). Brain-reactive autoantibodies are nearly ubiquitous in human sera and may be linked to pathology in the context of blood-brain barrier breakdown. *Brain Research*.

[20] Vacirca D., Barbati C., Scazzocchio B. (2011). Anti-ATP synthase autoantibodies from patients with Alzheimer's disease reduce extracellular HDL level. *Journal of Alzheimer's Disease*.

[21] Colasanti T., Barbati C., Rosano G., Malorni W., Ortona E. (2010). Autoantibodies in patients with Alzheimer's disease: pathogenetic role and potential use as biomarkers of disease progression. *Autoimmunity Reviews*.

[22] Neff F., Wei X., Nölker C., Bacher M., Du Y., Dodel R. (2008). Immunotherapy and naturally occurring autoantibodies in neurodegenerative disorders. *Autoimmunity Reviews*.

[23] Paul S., Planque S., Nishiyama Y. (2010). Immunological origin and functional properties of catalytic autoantibodies to amyloid *β* peptide. *The Journal of Clinical Immunology*.

[24] Hoogenboom H. R., de Brune A. P., Hufton S. E., Hoet R. M., Arends J.-W., Roovers R. C. (1998). Antibody phage display technology and its applications. *Immunotechnology*.

[25] Rader C., Barbas C. F. (1997). Phage display of combinatorial antibody libraries. *Current Opinion in Biotechnology*.

[26] Kriplani U., Kay B. K. (2005). Selecting peptides for use in nanoscale materials using phage-displayed combinatorial peptide libraries. *Current Opinion in Biotechnology*.

[27] Altschul S. F., Madden T. L., Schäffer A. A. (1997). Gapped BLAST and PSI-BLAST: a new generation of protein database search programs. *Nucleic Acids Research*.

[28] Huang J., Ru B., Dai P. (2011). Bioinformatics resources and tools for phage display. *Molecules*.

[29] Mayrose I., Shlomi T., Rubinstein N. D. (2007). Epitope mapping using combinatorial phage-display libraries: a graph-based algorithm. *Nucleic Acids Research*.

[30] American Psychiatric Association (2000). *Diagnostic and Statistical Manual of Mental Disorders: DSM-IV-TR*.

[31] McKhann G., Drachman D., Folstein M., Katzman R., Price D., Stadlan E. M. (1984). Clinical diagnosis of Alzheimer's disease: report of the NINCDS-ADRDA work group under the auspices of Department of Health and Human Services Task Force on Alzheimer's disease. *Neurology*.

[32] Montaño M., Ramos L. R. (2005). Validade da versão em português da clinical dementia rating. *Revista de Saúde Pública*.

[33] Folstein M. F., Folstein S. E., McHugh P. R. (1975). ‘Mini-mental state’. A practical method for grading the cognitive state of patients for the clinician. *Journal of Psychiatric Research*.

[34] Reis C. F., Carneiro A. P., Vieira C. U. (2013). An antibody-like peptide that recognizes malignancy among thyroid nodules. *Cancer Letters*.

[35] Larsen J. E., Lund O., Nielsen M. (2006). Improved method for predicting linear B-cell epitopes. *Immunome Research*.

[36] Ansari H. R., Raghava G. P. (2010). Identification of conformational B-cell Epitopes in an antigen from its primary sequence. *Immunome Research*.

[37] Arap M. A. (2005). Phage display technology—applications and innovations. *Genetics and Molecular Biology*.

[38] Aina O. H., Liu R., Sutcliffe J. L., Marik J., Pan C.-X., Lam K. S. (2007). From combinatorial chemistry to cancer-targeting peptides. *Molecular Pharmaceutics*.

[39] Vodnik M., Zager U., Strukelj B., Lunder M. (2011). Phage display: selecting straws instead of a needle from a haystack. *Molecules*.

[40] Manhani M. N., Ribeiro V. S., Cardoso R., Ueira-Vieira C., Goulart L. R., Costa-Cruz J. M. (2011). Specific phage-displayed peptides discriminate different forms of neurocysticercosis by antibody detection in the serum samples. *Parasite Immunology*.

[41] da Silva Ribeiro V., Manhani M. N., Cardoso R., Vieira C. U., Goulart L. R., Costa-Cruz J. M. (2010). Selection of high affinity peptide ligands for detection of circulating antibodies in neurocysticercosis. *Immunology Letters*.

[42] Goulart L. R., Vieira C. U., Freschi A. P. P. (2010). Biomarkers for serum diagnosis of infectious diseases and their potential application in novel sensor platforms. *Critical Reviews in Immunology*.

[43] Qu B.-X., Gong Y., Moore C. (2014). Beta-amyloid auto-antibodies are reduced in Alzheimer’s disease. *Journal of Neuroimmunology*.

[44] Du Y., Wei X., Dodel R. (2003). Human anti-*β*-amyloid antibodies block *β*-amyloid fibril formation and prevent *β*-amyloid-induced neurotoxicity. *Brain*.

[45] Wang X.-P., Zhang J.-H., Wang Y.-J. (2009). Conformation-dependent single-chain variable fragment antibodies specifically recognize beta-amyloid oligomers. *FEBS Letters*.

[46] Richens J. L., Morgan K., O'Shea P. (2014). Reverse engineering of Alzheimer's disease based on biomarker pathways analysis. *Neurobiology of Aging*.

[47] Ray M., Zhang W. (2010). Analysis of Alzheimer's disease severity across brain regions by topological analysis of gene co-expression networks. *BMC Systems Biology*.

[48] Drees F., Gertler F. B. (2008). Ena/VASP: proteins at the tip of the nervous system. *Current Opinion in Neurobiology*.

[49] Urbanelli L., Massini C., Emiliani C., Orlacchio A., Bernardi G. (2006). Characterization of human Enah gene. *Biochimica et Biophysica Acta—Gene Structure and Expression*.

[50] Franco D. L., Rezával C., Cáceres A., Schinder A. F., Ceriani M. F. (2010). ENA/VASP downregulation triggers cell death by impairing axonal maintenance in hippocampal neurons. *Molecular and Cellular Neuroscience*.

[51] Trillo L., Das D., Hsieh W. (2013). Ascending monoaminergic systems alterations in Alzheimer's disease. Translating basic science into clinical care. *Neuroscience and Biobehavioral Reviews*.

[52] Pennington P. R., Wei Z., Rui L. (2011). Alzheimer disease-related presenilin-1 variants exert distinct effects on monoamine oxidase-A activity in vitro. *Journal of Neural Transmission*.

[53] Rogelj B., Mitchell J. C., Miller C. C. J., McLoughlin D. M. (2006). The X11/Mint family of adaptor proteins. *Brain Research Reviews*.

[54] Chaufty J., Sullivan S. E., Ho A. (2012). Intracellular amyloid precursor protein sorting and amyloid-*β* secretion are regulated by Src-mediated phosphorylation of Mint2. *Journal of Neuroscience*.

[55] Matos M. F., Xu Y., Dulubova I. (2012). Autoinhibition of Mint1 adaptor protein regulates amyloid precursor protein binding and processing. *Proceedings of the National Academy of Sciences of the United States of America*.

[56] Lee J.-H., Lau K.-F., Perkinton M. S. (2003). The Neuronal Adaptor Protein X11*α* Reduces A*β* Levels in the Brains of Alzheimer's APPswe Tg2576 Transgenic Mice. *The Journal of Biological Chemistry*.

[57] Maina F., Klein R. (1999). Hepatocyte growth factor, a versatile signal for developing neurons. *Nature Neuroscience*.

[58] Tsuboi Y., Kakimoto K., Nakajima M. (2003). Increased hepatocyte growth factor level in cerebrospinal fluid in Alzheimer's disease. *Acta Neurologica Scandinavica*.

[59] Fenton H., Finch P. W., Rubin J. S. (1998). Hepatocyte growth factor (HGF/SF) in Alzheimer's disease. *Brain Research*.

[60] Cullen P. J. (2008). Endosomal sorting and signalling: an emerging role for sorting nexins. *Nature Reviews Molecular Cell Biology*.

[61] Huang H.-S., Yoon B.-J., Brooks S. (2014). *Snx14* regulates neuronal excitability, promotes synaptic transmission, and is imprinted in the brain of mice. *PLoS ONE*.

[62] Mizutani R., Nakamura K., Kato N. (2012). Expression of sorting nexin 12 is regulated in developing cerebral cortical neurons. *Journal of Neuroscience Research*.

[63] Zhao Y., Wang Y., Yang J., Wang X., Zhang X., Zhang Y.-W. (2012). Sorting nexin 12 interacts with BACE1 and regulates BACE1-mediated APP processing. *Molecular Neurodegeneration*.

[64] Wang X., Zhao Y., Zhang X. (2013). Loss of sorting nexin 27 contributes to excitatory synaptic dysfunction by modulating glutamate receptor recycling in Down's syndrome. *Nature Medicine*.

[65] Li B., Yu D., Xu Z. (2014). Activated protein C inhibits amyloid *β* production via promoting expression of ADAM-10. *Brain Research*.

[66] Guo H., Liu D., Gelbard H. (2004). Activated protein C prevents neuronal apoptosis via protease activated receptors 1 and 3. *Neuron*.

[67] Oku Y., Huganir R. L. (2013). AGAP3 and Arf6 regulate trafficking of AMPA receptors and synaptic plasticity. *Journal of Neuroscience*.

[68] Nagashima S., Fukuda T., Kubota Y. (2011). CRMP5-associated GTPase (CRAG) protein protects neuronal cells against cytotoxicity of expanded polyglutamine protein partially via c-fos-dependent activator protein-1 activation. *The Journal of Biological Chemistry*.

[69] Nagele E., Han M., DeMarshall C., Belinka B., Nagele R. (2011). Diagnosis of Alzheimer's disease based on disease-specific autoantibody profiles in human sera. *PLoS ONE*.

